# Loneliness in University Students during Two Transitions: A Mixed Methods Approach Including Biographical Mapping

**DOI:** 10.3390/ijerph20043334

**Published:** 2023-02-14

**Authors:** Janna Jaud, Tatiana Görig, Tobias Konkel, Katharina Diehl

**Affiliations:** 1 Department of Medical Informatics, Biometry and Epidemiology, Professorship of Epidemiology and Public Health, Friedrich-Alexander-Universität Erlangen-Nürnberg, 91054 Erlangen, Germany; 2 Institute for Medical Information Processing, Biometry and Epidemiology—IBE, LMU Munich, 81377 Munich, Germany; 3 Pettenkofer School of Public Health, 81377 Munich, Germany

**Keywords:** loneliness, transition, students, university, COVID-19

## Abstract

Several studies have shown that loneliness is prevalent in university students. However, up to now, it is less clear how transitions during this life stage are associated with loneliness. Therefore, we aimed to explore the association of loneliness with the transition from high school to university and the transition into the COVID-19 pandemic. Twenty students were interviewed in qualitative interviews based on a semi-structured guide that also included biographical mapping. In addition, the participants reported social and emotional loneliness based on the six-item De Jong Gierveld Loneliness Scale for three points in time: (1) at the time of the interview, (2) at the beginning of their studies at the university and (3) at the start of the COVID-19 pandemic. The qualitative data were analyzed using a structuring content analysis following Mayring. The quantitative data were analyzed using descriptive statistics. We found that emotional loneliness increased both during high school graduation and at the start of study at the university, as well as at the beginning of the COVID-19 pandemic. Social loneliness was higher during university studies than during the last years at high school and increased at the beginning of the pandemic. The results indicate that both transitions played an important role for perceived social and emotional loneliness. Further quantitative studies in larger samples will be relevant in the future to better target the responses to loneliness during transitions. Universities can actively counteract loneliness, especially during the transition from high school to university, by organizing events and meeting places where new students can network.

## 1. Introduction

Loneliness is a relevant public health-related topic at all ages, as it can have effects on people’s mental and physical health [1,2,3]. For example, a systematic review has shown that loneliness is associated with all-cause mortality and cardiovascular diseases [2]. While loneliness has been studied among older people for decades, loneliness among young people has not been present in research for long [4,5,6]. One specific target group among young people are university students who have been shown to be at risk for loneliness and social isolation in previous studies [7,8,9,10,11,12].

Life events such as transitions are known to have an impact on health, health behaviors, as well as the perceptions of loneliness. An example is the school-to-work transition, which was shown to be related to health and well-being [13]. However, transitions that are not anticipated, such as divorce, separation and widowhood, can also have effects on health [14]. One specific transition is the transition from high school to university, which is handled by millions of young people worldwide each year. The transition from high school to university is associated with structural, social, psychological and financial changes [15]. Oftentimes, the start of study is related to a change in the place of residence. Associated with this, young people have to build up a new circle of friends and need to learn to live a life that is different from their familiar high school life [11]. Previous studies have shown that transitions from high school to university can result in changes in the students’ health-related risk behaviors and health [16,17,18].

The outbreak of the COVID-19 pandemic in the spring of 2020 represented a transition into a completely unfamiliar and very special situation—not only for university students. Here, a transition took place from the usual everyday life into a time characterized by lockdowns and uncertainty. These lockdowns were shown to be associated with changes in health, health behaviors and loneliness in the general population, but also in students [10,19,20,21].

As in many other countries, the first lockdown in Germany due to COVID-19 was declared in March 2020. Strict contact restrictions that brought serious changes to education both in schools and universities were put in place in Germany. For example, face-to-face teaching for students was no longer possible and was replaced with online/remote classes. Students, who had started studying in the spring or winter semester in 2020, could not attend any in-person course at their university for up to one and a half years. Previous studies have shown that the mental health of students worsened during this time and that loneliness increased [21,22,23]. For instance, a recent study among students at a German university [24] revealed an increase in loneliness as well as a slight increase in depression when comparing the pre-pandemic values to those during the COVID-19 pandemic. 

According to Weiss [25], loneliness can be divided into two categories. On the one hand, there is social loneliness, which describes the lack of a social network of friends, acquaintances and colleagues with whom one spends their leisure time [25]. On the other hand, there is emotional loneliness, which describes the absence of a close person of trust and thus the absence of a deep and meaningful relationship [25]. It is conceivable that both forms of loneliness occurred in university students during the transition from high school to university and during the COVID-19 pandemic.

The aim of our study was therefore to investigate the trajectories in social and emotional loneliness among university students, including two transitions: (1) the transition from high school to university and (2) the transition from pre-COVID-19 into the COVID-19 pandemic. To visualize the potential changes in loneliness in the course of time, we used the innovative method of biographical mapping [11,16]. In addition, we quantitatively assessed social and emotional loneliness at the time of the interview as well as retrospectively at the start of study and the start of the COVID-19 pandemic. This enabled us to not only describe loneliness at three points in time but also helped to validate the results yielded by biographical mapping. Moreover, we collected the data using a semi-structured interview guide. Based on the qualitative interviews, we were able to better understand the development of loneliness over time and provide additional and explanatory information on the trajectories of loneliness during the two above-named transitions.

## 2. Materials and Methods

### 2.1. Study Population

To learn more about the trajectories of loneliness among university students, we conducted a qualitative study, including biographical mapping [16] and a quantitative component. Twenty interviews were conducted with students in Southern Germany between November 2021 and February 2022. The participants were recruited as a convenient sample through the university newsletter, a youth association and the personal contacts of the first author (J.J.). The inclusion criteria to participate in the interview were a sufficient knowledge of German, having started studying directly after high school graduation, current university enrollment and being at least in the fifth study semester. These criteria allowed us to compare the data of biographical mapping more easily. A gap year or voluntary social year was an exclusion criterion due to the difficulty in comparing the trajectories. 

Prior to the interviews, all the participants were informed about the data protection and the processing of their data. They were also informed that their participation was voluntary and that consent could be withdrawn at any time. All the subjects gave their written consent to participate in the study, as well as for the data collection and processing. As reimbursement, all the participants received a EUR 15 gift voucher. The study was approved by the Ethics Committee II of the Medical Faculty Mannheim, Heidelberg University on 16 November 2021 (number 2021-652).

### 2.2. Measures

The study was designed as a qualitative study, which included the collection of sociodemographic information (e.g., age, sex, current living situation) and study-related aspects (e.g., kind of university, Bachelor/Master program, semester), and a quantitative aspect as well as biographical mapping. The collection of the quantitative and qualitative data, as well as the biographical mapping, took place during a single appointment. The data collection was carried out in the form of semi-structured guided interviews that were conducted face-to-face by the first author J.J. (female, B.A. in Social Work). J.J. was intensively trained by the last author K.D., an experienced interviewer. Prior to the first interview, J.J. conducted a test interview. The interviews lasted for an average of 1 h and 6 min (min. 33 min, max. 1 h and 47 min). The interviews took place in locations determined by the participants themselves where they felt comfortable and free to talk. 

#### 2.2.1. Quantitative Component

Before starting with the qualitative interview, the quantitative study component took place. In this study component, we assessed the current status of emotional and social loneliness as well as the retrospective status at two other time points: (1) the start of study and (2) the start of the COVID-19 pandemic and its associated first lockdown in spring 2020. We used the six-item De Jong Gierveld Loneliness Scale [26,27] consisting of the following six items.

Emotional subscale:

1. I experience a general sense of emptiness.
2. I miss having people around.
3. I often feel rejected.

Social subscale:

1. There are plenty of people I can rely on when I have problems.
2. There are many people I can trust completely.
3. There are enough people I feel close to.

The participants could rate each of these items on a four-point scale (“strongly agree”, “agree”, “disagree”, “strongly disagree”) [11]. For processing the data, the positive answers on the emotional subscale items were counted, resulting in the emotional loneliness score ranging from zero to three, with zero corresponding to being “not emotionally lonely” and three indicating “intensely emotionally lonely” [26,27]. Based on the negative answers on the social subscale items, the social loneliness score was formed. It also ranged from zero (“not socially lonely”) to three (“intensely socially lonely”) [26,27]. There were no missing values. An overall loneliness score was calculated by combining the scores of social and emotional loneliness, ranging from 0 to 6 (0–1 = “not lonely”, 2–4 = “moderately lonely”, 5–6 = “severely lonely”).

#### 2.2.2. Qualitative Data

After the quantitative assessment, the semi-structured interviews took place. The interview guide started with questions on loneliness in general. Afterward, the definition of social and emotional loneliness following Weiss [25] was presented to the participants. It was discussed with the participants whether this distinction made sense from their point of view based on their subjective experience. Following this, the interviews focused on emotional and social loneliness during the transition from high school to university and the transition into the COVID-19 pandemic. The sub-aspects were the perceived influence of the change of residence for the start of study and loneliness. Regarding both transitions, the sources of support, available resources and desired support regarding loneliness were thematized.

To qualitatively analyze the interviews, a total of 40 main codes and 170 subcodes were used to adequately describe the participants’ statements. For the findings presented in this manuscript, the codes related to “loneliness in general”, “loneliness during the transition from high school to university” and “loneliness during the COVID-19 pandemic” were used.

#### 2.2.3. Biographical Mapping

Subsequently, the biographical mapping method [16] was introduced to the participants. This method makes it possible to graphically represent individual courses in life. It helps to get an idea whether a behavior or a status is stable or whether there is variation over time. This enabled us, for example, to assess loneliness in more detail than in the quantitative surveys. In addition, the participants had the opportunity to better reflect on their perceived loneliness retrospectively.

A mapping grid was used for this purpose, in which the x-axis reflected a time course, starting from 2014, while the y-axis represented the perceived intensity of social and emotional loneliness on a scale ranging from 0 (very low) to 10 (very high). Using vertical lines, the two transitions were highlighted in the grid.

**Figure 1:**
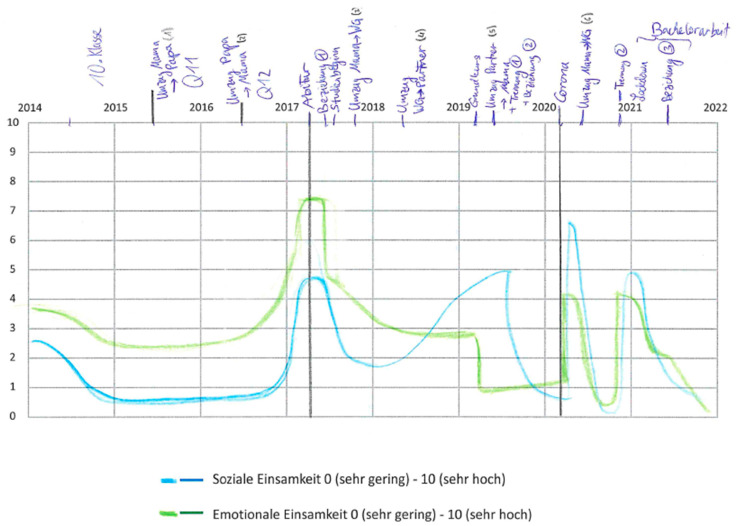
Example of a filled in mapping grid. For the data analysis, the x-axis values for each quarter of a year measured by a conventional ruler were transferred.

The mapping grid was printed on an A4 sheet. As a first step, the respondents were asked to identify the educational milestones in their lives and mark the timepoints in the mapping grid (e.g., Abitur certificate, start of study, Bachelor’s degree). After that, they were asked to highlight additional important life events (e.g., end of relationship, new relationship, death of a relative). Once all the important events were drawn in the map, the participants used different colored pens—blue for social loneliness and green for emotional loneliness—to draw the trajectories over time. For an example of a filled in grid, see Figure 1.

### 2.3. Data Analysis

All the interviews were audio-taped with a recording device (Olympus WS-853) and transcribed verbatim. In order to analyze the transcripts, a qualitative content analysis according to Mayring [28] was used. To systemize the data, the categories and subcategories were identified based on the semi-structured interview guide. Accordingly, codes were developed and constantly revised during the course of the analysis. For coding the data, we used the program MAXQDA (VERBI GmbH, Berlin, Germany, Version 18.2.0).

The six-item De Jong Gierveld Scale on emotional and social loneliness was analyzed using IBM SPSS statistics (IBM, Armonk, Version 28). The six-item scale was coded as suggested by the inventors, resulting in two scores, one for social and one for emotional loneliness ranging each from zero to three (see Section 2.2.1). In addition, the overall loneliness (range 0–6) was reported. For description, we calculated the median, mean and standard deviation for both social and emotional loneliness and for the overall loneliness. To compare loneliness at start of study and at the start of the COVID-19 pandemic with the current loneliness, we calculated a Wilcoxon signed-rank test. A *p*-value < 0.05 was considered as significant.

To analyze the biographical mapping (Figure 1), a conventional ruler was used to identify the values at the x-axis (i.e., the perceived intensity of social and emotional loneliness) for each quarter of a year. In order to be able to describe the trajectories, the data for every participant were transferred into SPSS. The findings were displayed in figures reporting the mean and standard deviation.

## 3. Results

Twenty students participated in this study. The mean age of the participants was 24.1 (SD: 1.6) years. Twelve of the students surveyed were female (60%). Fifteen participants changed the place of their residence at least once in order to study (75%). The mean number of semesters studied in their current program was 3.5 (SD: 2.1). Fifteen students were enrolled in a master’s degree program, four in a bachelor’s degree program (20%) and one student in state examination program (“Staatsexamen”) (5%, Table 1). For the most part, the students were involved in extracurricular activities (85%). The majority lived in a shared apartment (75%). Six out of the ten participants were in a relationship at the time of interviewing (60%). The detailed characteristics of the study sample are shown in Table 1.

### 3.1. Quantitative Component

**Figure 2:**
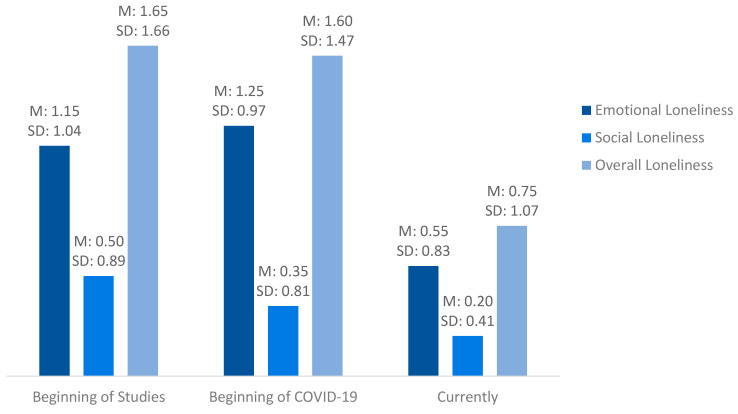
Loneliness in the participating students at three time points. Data for the time points “Beginning of study” and “Beginning of COVID-19” were assessed retrospectively; Data were collected via the six-item De Jong Gierveld Loneliness Scale. M: mean, SD: standard deviation, emotional and social loneliness score: min: 0, max: 3, overall loneliness score: min: 0, max.: 6; n = 20.

Loneliness based on the six-item De Jong Gierveld Loneliness Scale [26,27] was assessed for three points in time (Figure 2). Although the extents of emotional, social and the overall loneliness were relatively low, the mean values were higher at the beginning of study and the beginning of the COVID-19 pandemic than at the time of the interview (i.e. currently). The Wilcoxon signed-rank tests revealed that the difference of emotional loneliness between the current status and the two starting points of transition were significant (*p* = 0.018 for the beginning of study vs. currently; *p* = 0.042 for the beginning of the COVID-19 pandemic vs. currently). In addition, the overall loneliness score differed significantly between the beginning of study and the current status (*p* = 0.007), while there was a tendency for the comparison between the beginning of the COVID-19 pandemic and the current status (*p* = 0.055).

Overall, three out of the 20 (15%) participants felt severely emotionally lonely at the beginning of study, two out of the 20 (10%) at the beginning of the COVID-19 pandemic and none currently felt severely emotionally lonely. Regarding social loneliness, one out of the 20 (5%) felt severely lonely at the beginning of study and one out of the 20 (5%) felt lonely at the beginning of pandemic, while none of the students currently felt severely socially lonely.

### 3.2. Biographical Mapping

**Figure 3:**
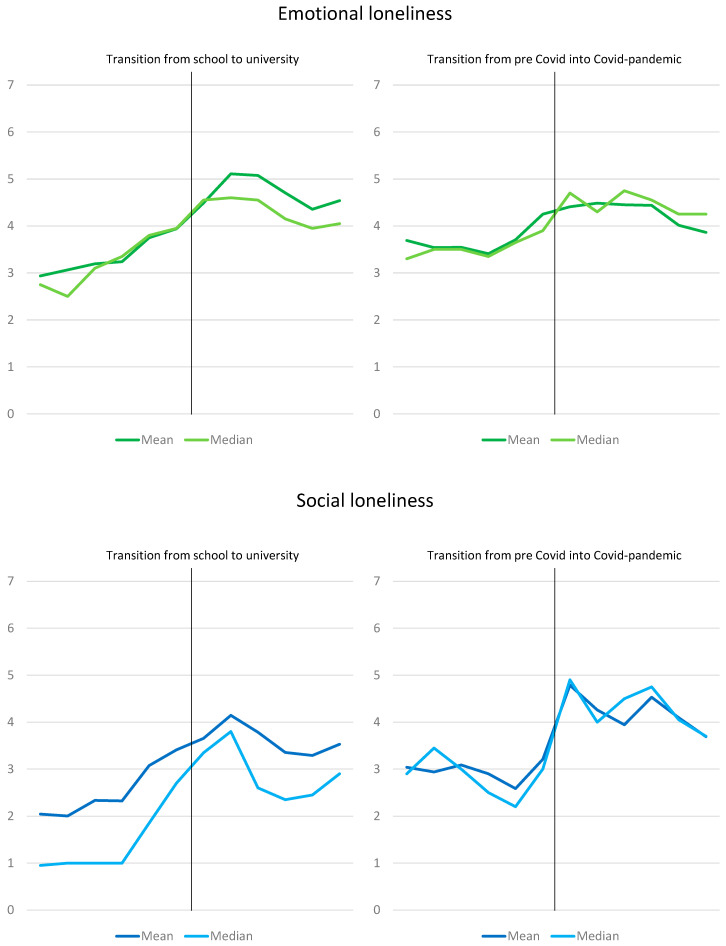
Trajectories of loneliness in the participating students based on biographical mapping. Range of loneliness: 0–10, n = 20.

The data of the biographical mapping were transferred into the mean values of the study population for each quarter of a year (Figure 3). Overall, emotional loneliness had higher values than social loneliness except at the start of the COVID-19 pandemic, where social loneliness showed a peak (mean: 4.8, median: 4.9).

Emotional loneliness did not only increase after the transition from high school to university, but the increase already started during the preparation for the German “Abitur” certificate. The beginning of the COVID-19 pandemic was also associated with an increase in emotional loneliness. Apart from the changes in the trajectories around the two transition points, the variations based on the individual events, such as moving, death of a close relative, or separation from a boyfriend/girlfriend, could be identified. The same applied to social loneliness.

Social loneliness was lower during high school compared to the time at university (post-transition and pre-COVID-19 situation; Figure 3). However, the trajectories showed an increase in social loneliness during the preparation for the “Abitur” certificate prior to the transition to university. The increase in social loneliness after the beginning of the COVID-19 pandemic was steeper than for emotional loneliness.

### 3.3. Qualtiative Findings

In the following, the findings of the semi-structured interviews will be presented. First, we will shortly describe the students’ general view of loneliness and the definition of loneliness (Section 3.3.1). Then we provide the findings on loneliness during the transition from high school to university (Section 3.3.2) and the impact of the change of residence on loneliness (Section 3.3.3). Finally, we present our findings regarding loneliness during the COVID-19 pandemic (Section 3.3.4). In brackets, the IDs of the participants are reported (e.g., S01).

#### 3.3.1. Students’ General View of Loneliness

All the students reported to have already experienced loneliness without defining it more precisely. Eighteen of the twenty participants associated the feeling of loneliness with being alone. However, the feeling of being misunderstood by significant others was also perceived as associated with the term loneliness (S07, S08, S11, S13, S15, S16). S11 described it as “*Loneliness is the feeling I have when I’m left alone with problems and don’t know whom to contact at that moment*”. While the majority defined loneliness as a negative feeling, one participant also found something positive in self-imposed loneliness (S20).

In general, loneliness was mainly experienced on weekends without social contacts, during periods of exams or during disagreements with significant others. Loneliness was particularly pronounced during the separation from a partner (S03, S15, S17, S18).

The distinction between social and emotional loneliness made sense to nineteen students. Many of these had already tried to fit their experiences of loneliness into similar categories before becoming familiar with the distinction. Several participants, however, noted that they would not consider both types separately, but rather considered some of the boundaries to be blurred. Only one participant considered the distinction as not meaningful, because, for him, both forms were completely related (S14).

Social loneliness was experienced by the respondents primarily in young adulthood, predominantly during transitions, but was also an issue during the high school years (S03, S05, S06, S08). Emotional loneliness was increasingly experienced due to a lack of understanding or friendships drifting apart. However, the participants reported less frequently about situations in which they perceived emotional loneliness.

#### 3.3.2. Loneliness and the Transition from High School to University

The start of study was described by the participants as exciting and as a time in which many new things were experienced (S06, S07, 10, S13, S14, S15, S16, S17). New contacts were quickly sought and also found (S03, S06, S07, S08, S09, S12, S13, S16, S17). In some cases, however, the students found it difficult to adapt from the usual demands of high school to those of the university, which were sometimes perceived as overwhelming (S02, S03, S10, S18, S19). For instance, S19 told the interviewer: “*Yes, I found it very exciting, but also overwhelming, very overwhelming. So, I felt like I was zero prepared to go to university*”.

The excessive demands, however, might also have their origin in the lack of preparation for university during the last years at high school. Many participants reported that they felt inadequately prepared by their high school for their university studies (S01, S05, S07, S08, S09, S10, S13, S18, S19, S20). Seven respondents even stated that they were not prepared at all (S02, S03, S04, S11, S12, S14, S17). Only two participants felt that high school prepared them adequately for their studies in terms of the content or subject matter (S06, S15). Some of them stated that they took part in career orientation seminars in high school, but that these seminars were not considered to be helpful (S10, S18).

To help the students getting started with their studies, many universities offer support services during the first semester. However, these offers seem to differ greatly between German universities. Almost every participant reported an introductory week for first semester students. The interviewees praised these events very much, as it allowed them to quickly find new contacts and feel less alone in the new and sometimes overwhelming situations (S01, S04, S05, S07, S08, S10, S12, S13, S15, S16, S17, S18). The other important possibility to make contacts were the student councils. These councils organized many events that helped the students make new acquaintances outside of lectures. However, some interviewees reported that alcohol played a great role in many of these get-to-know events (S02, S03, S10, S11, S12, S15, S16). The people who drank little or no alcohol felt very uncomfortable at such events or deliberately avoided visiting these. For instance S16 said: “*I think, at that time, I didn’t drink either and then somehow it was a bit too much alcohol for me*”. Others indicated that they perceived these events as very liberating to party and let loose without the control of their parents.

For many students, social loneliness was more pronounced during the transition from high school to university, because although they had people around them, they did not (yet) know enough of them and, therefore, felt restricted from engaging in social activities from the beginning (S02, S03, S04, S06, S08, S10, S14). S03 chose the following words to describe perceived social loneliness: “*There was already a great social loneliness, I would say. It was simply the fact that there was (…) no environment in which you could simply do something, exchange ideas, have a bit of fun and so on. That was simply missing”*. Nevertheless, some students reported experiencing emotional loneliness at the start of their studies because they had not yet had time to build deeper friendships (S01, S10, S13, S20).

#### 3.3.3. Change of Residence at the Start of Study and the Association with Loneliness

Fifteen students changed their residence for starting their studies. Nine out of the fifteen students regarded this change as a reason for loneliness at the start of their studies (S01, S02, S03, S08, S10, S11, S12, S13, S19). It was mostly experienced after the courses and the participants did not know how to keep themselves busy (S01, S08).

Overall, the change of residence was experienced in a mixed way. While eight students regarded it as a relief and were looking forward to starting a new life and becoming more independent (S05, S06, S10, S13, S16, S17, S20), three students described mixed feelings resonating with the move (S1, S8, S13). For example, S13 stated: “*I honestly wanted to get as far away as possible at the beginning, so I found it exciting at first, but yes, in any case, I also felt a bit lost*”. Five participants described the change of residence as very difficult (S02, S03, S04, S11, S12).

#### 3.3.4. Loneliness during the Transition into the COVID-19 Pandemic

The transition to the COVID-19 pandemic and the drastic changes it brought to the daily lives of students were experienced as a great burden by some of the interviewees. The focus was on the changes brought by the first lockdown in spring 2020.

The reasons for the perceived changes in loneliness were manifold. The most important change concerned the fact that meeting family members and significant others was restricted (S01, S02, S07, S08, S09, S12, S14), resulting in a feeling of isolation from friends and the outside world (S10, S19, S20). The students were very frustrated by this change and the contact restrictions (S01, S06, S08, S12, S14, S18). For some participants, the loss of sport activities was also relevant and their motivation to continue to engage in sports decreased (S01, S02, S03, S09, S12). In addition, the time was experienced as very exhausting (S01, S09, S10, S11, S12, S14, S19).

However, there were also students who did not notice any or at least very little change to their everyday life. The reasons given for this were a job alongside their studies or having already hardly any social contacts due to the completion of a final thesis (S03, S06, S07). Seven students stated that they perceived the first period of the pandemic as relaxing and that the restrictions had not affected them negatively (S03, S07, S08, S13, S17, S18, S20). S17 reported, for example: “*(…) well, at first I actually enjoyed it a little bit, because a little bit of calm came into all this hectic pace, so to speak*”.

The biggest change in the university setting for mainly all the participants was that all the lectures and seminars were first cancelled and then held online (S01, S02, S04, S05, S07, S08, S12, S13, S14, S15, S16, S17, S18, S19, S20). This resulted in a loss of social contacts (S01, S07, S08, S12, S13, S14, S15, S16, S17, S19). For the students who did not have a strong social network before the pandemic began, it became more difficult to establish contacts with fellow students, make friends and, therewith, counteract the threat of loneliness.

The longer the courses were held online, the more stressful it became for many respondents: “*it’s just super exhausting to sit in front of the computer all day, so it’s like you get a little bit tired, or you lose your motivation in life a little bit (…)*” (S19). Those who started a master’s program during the pandemic had no personal contact with their fellow students or did not even get to know them for two to three semesters (S01, S13, S14, S17, S18).

*“I lost contact with friends. I simply did not meet anymore due to the contact restrictions”* was reported by S08, which illustrated the main reason for social loneliness in many participants (S01, S02, S03, S05, S06, S07, S08, S10, S12, S14, S15, S16, S18, S19, S20). In addition, friendships became less intense (S07, S08, S12, S14, S16).

Emotional loneliness was mainly reflected through limiting contact with significant others, such as friends and family (S01, S04, S05, S07, S08, S09, S15, S17, S18). Physical closeness and deep conversations were missing. The negative news and the isolation affected the mood of some students (S06, S09, S14). The participants reported that when they met others to overcome perceived loneliness, they felt guilty, even if it was not against the legal restrictions (S03, S15, S16). The effects on mental health were also noticed by some participants, e.g. by S07: “*For me it simply manifested itself in the fact that I wasn’t doing well. So, the lack of contact to especially fellow students or people who had the same problems as me (…). Well, I felt lonely and no longer had the motivation to get up, to work, to do my things*”.

During this time, support from the university might have been very important to many students, yet eleven of the participants indicated that there were no supportive offers from the university at all (S02, S03, S06, S09, S10, S11, S12, S13, S14, S15, S20). At least some interviewees shared offers for consultations via mailing lists. However, this was usually the only assistance provided by the university (S01, S05, S09, S11, S17, S19). If there were further offers or exchanges, these tended to be organized by students or the student council, not by the university (S01, S07, S08, S10, S16, S18).

#### 3.3.5. Attempts to Overcome Loneliness during the COVID-19 Pandemic

Seven students moved back to their parents during the pandemic, either because they could not go on with their original plans or due to contact restrictions since they did not want to be alone (S01, S02, S03, S10, S12, S19, S20). It showed that the housing situation led to major differences in the perception of loneliness. Those who lived in a shared apartment at the time of the first lockdown subjectively suffered less from the contact restrictions and also named the housing situation as a protective factor (S04, S05, S06, S10, S11, S13, S15, S16): “*Moving into a shared apartment helped. That really saved me a lot, I think. So, the last winter would have been much worse, I think, if I had not been here in the shared apartment*” (S16).

Many students reported that it was helpful to maintain contact with parents and family (S01, S06, S10, S11, S12, S14, S15, S20) and to have a regular exchange with friends (S01, S02, S03, S06, S09, S11, S12, S15, S16, S18, S19), for instance, during walks, phone calls, or online game nights. Physical activity was an important measure against loneliness for many respondents, especially if they were able to leave their homes for the activity (S01, S02, S03, S04, S08, S11, S12, S13, S19). Another important factor was that daily routines were established or maintained. Since there was little variety and a lot of time in front of the screen, it was important for students to stick to daily patterns in order not to lose a certain structure (S02, S05, S06, S09, S12, S18, S19).

### 3.4. Synopsis

Overall, the results from the three data components fit well together. The quantitative element as well as the biographical mapping suggested a relationship of the two transitions with loneliness. This was supported by the qualitative data.

In the quantitative loneliness scale, emotional loneliness scored higher than social loneliness at both transition points. This was also reflected in the biographical mapping. The sole exception was the time point directly after the start of the COVID-19 pandemic, where social loneliness was higher than emotional loneliness. At the beginning of study, social loneliness was reasoned in the qualitative interviews by the fact that the students had not yet found many new acquaintances. The explanation for the perceived emotional loneliness was from the students’ point of view that deep friendships had not yet developed. During the COVID-19 pandemic, limited contacts were described as leading to both social and emotional loneliness.

## 4. Discussion

Our study included three components—qualitative interviews, a quantitative data element and biographical mapping—with the aim of exploring whether loneliness in university students was related to transitions. We found that the transition from high school to university, as well as the transition into the COVID-19 pandemic, were associated with the perception of loneliness among the participants. Our data suggested that this applied to both social and emotional loneliness.

Using the six-item De Jong Gierveld Loneliness Scale [26,27] for two time points retrospectively and for the current situation illustrated that, during the time of high school graduation and the start of university as well as at the beginning of the COVID-19 pandemic, loneliness was significantly higher than at the time when the study was conducted. The biographical mapping also revealed that loneliness increased sharply and abruptly at these points in time and did not drop back to the initial level. These findings underscored the findings of the qualitative component.

An earlier study from Norway showed that loneliness among students has increased significantly from 2014 (16.5%) to 2018 (23.6%) [29]. However, no distinction was made between social and emotional loneliness. In contrast, a study from Germany conducted before the COVID-19 pandemic that revealed that 3.2% of students felt severely socially lonely and 7.7% felt severely emotionally lonely [11]. It must be clearly emphasized that these results represent the period before the COVID-19 pandemic. Several studies already showed that the situation has worsened since the pandemic [10,23,24]. In our study, none of the students currently felt severely lonely which may, however, be reasoned by our small sample size (n = 20).

### 4.1. Transition from High School to University

For many students, the transition from high school to university is synonymous with a start into a new life. Many students move to a new city without an existing circle of friends or a network [15,30]. We know from previous studies that this transition, and especially the time before the transition, can cause stress [18] and is a critical period in life [31].

The participants in this study found the time of transition mostly exciting, but also overwhelming. None of the interviewees felt adequately prepared by their high school for the start of their university studies. However, the participants reported that the universities were able to pick up and support many of them at the start of their studies. The so-called introductory weeks for freshmen and the events organized by the student council were described as a great help to quickly find new acquaintances and friends. These organized events might be an important step to counter the emergence of loneliness. Since several studies also show that the first year at university in particular leads to a deterioration in mental health, including loneliness, for many students, this point is very relevant [18,32].

During the transition from high school to university, social loneliness was perceived by many participants. This seems unsurprising due to the aforementioned change of residence and the new environment [15]. New social relationships take time to be established. At the same time, students have to take responsibility for themselves, which is a new experience for most of them [15,30], exposing them to various demands.

### 4.2. Transition into the COVID-19 Pandemic

Several studies have provided evidence that the COVID-19 pandemic, accompanied with the restrictions on daily life, was associated with loneliness and the feeling of isolation in the general population [10,33]. The impact of the lockdown(s) on health is currently the subject of intensive research. However, it is already apparent that the lockdowns had an overall negative impact on mental health [33,34] in both the general population [35] and university students [36]. For instance, a recent study from Germany showed that perceived lockdown stress and loneliness during the COVID-19 pandemic were associated with mental health in university students [24].

Our study showed that students hardly felt supported by their universities during this difficult time. They indicated that—apart from brief information about counselling possibilities—there was hardly any support. The poor flow of information causing stress for our participants was also criticized. Another study found that the new study conditions during the pandemic caused significantly more stress, which had a negative impact on health [37]. Weiss et al. [38] also showed that learning itself was also significantly more challenging during the pandemic. Matos Fialho et al. [37] concluded that more help from universities during the pandemic would have led to a lower negative impact of the pandemic on mental health, which was also reported by our participants.

In our study, some participants were unable to block out loneliness and isolation and moved back to their parents to feel less lonely. Shared housing was reported as a protective factor to counteract loneliness during the COVID-19 pandemic by our participants. In addition, many students were no longer able to afford an apartment or shared housing during the pandemic, as many typical student jobs in the service sector were no longer available [39,40]. Exchanges with friends via phone calls or even online services helped students to interact and feel less lonely during the lockdowns, although most emphasized that it was not an adequate substitute for face-to-face meetings.

### 4.3. Limitations

Our findings provide insights into loneliness during the transitions; however, they may be subject to some potential limitations. First, since the main data component (the semi-structured interviews with biographical mapping) followed a qualitative approach, the case number for the quantitative component was limited. Second, we used the six-item De Jong Gierveld Loneliness Scale [26,27] to assess loneliness in the current situation and retrospectively. This may have led to a recall bias. The individuals who perceived the situation as particularly bad may tend to overestimate loneliness retrospectively. Third, our sample consisted of a convenient sample mainly from universities in Bavaria (Southern Germany). Since we used the snowballing technique for the recruitment, there might be a bias leading to a more homogenous group of participants. In our case, there were more students enrolled in a Master’s program than in a Bachelor’s program, because our inclusion criterion was that students had to be at least in the 5th semester. This limitation was necessary to make good use of the biographical mapping. Therefore, the findings may not be generalizable to Germany, which was, however, not the primary aim of the qualitative studies. Fourth, loneliness is a very sensitive topic. Therefore, it might be that—due to social desirability—some students may not have been fully open to report on their perceived loneliness. This may lead to an underestimation in our study. Fifth, in our study, we focused only on emotional and social loneliness. However, also existential loneliness may exist in university students and should be investigated in future studies.

## 5. Conclusions

Our findings show that both the transition from high school to university and the transition into the COVID-19 pandemic were related to loneliness. The qualitative interviews revealed that universities are seen as key player for reducing particularly social loneliness through organizing events, mentoring and introductory weeks for students to get in contact with other students that are in the same situation.

The transition into the COVID-19 pandemic was associated with both the social and emotional loneliness of the students. The main reasons were the contact restrictions and predominantly online learning. Here, it was no longer possible to meet friends and acquaintances, which triggered loneliness in many students and caused some of them move back to their parents’ homes. Based on our findings, the students perceived that the universities did not provide adequate help during this challenging time.

Regarding future studies, our findings suggest that biographical studies with larger sample sizes should be conducted to identify in more detail at which points in time loneliness occurs or increases, which gives important hints for health promotion at universities. Our study showed that students in both transitions wished for more support from universities. Reducing loneliness among university students is an important aspect to foster health and to help students overcome the subsequent transitions with good health.

## Figures and Tables

**Table 1:** Description of the study participants (*n* = 20).

ID	Age of Participant, y	Sex of Participant	Change of Residence for Study	Number of Semesters in Current Program	Program Type	Participation in Extracurricular Activities	Current Housing Situation	Current Relationship Status
P01	23	Female	Yes	3	Master’s	Yes	shared apartment	in a relationship
P02	26	Male	Yes	9	State Examination	Yes	shared apartment	single
P03	26	Male	No	3	Master’s	Yes	shared apartment	single
P04	23	Male	Yes	7	Bachelor’s	Yes	shared apartment	in a relationship
P05	25	Female	Yes	1	Bachelor’s	Yes	shared apartment	single
P06	24	Male	Yes	3	Master’s	Yes	shared apartment	in a relationship
P07	24	Female	No	1	Master’s	Yes	living with parents	in a relationship
P08	24	Male	Yes	6	Master’s	Yes	shared apartment	in a relationship
P09	21	Female	Yes	1	Master’s	Yes	shared apartment	in a relationship
P10	25	Male	Yes	3	Master’s	Yes	shared apartment	single
P11	22	Female	Yes	1	Master’s	Yes	shared apartment	in a relationship
P12	24	Female	Yes	3	Master’s	Yes	shared apartment	in a relationship
P13	27	Female	Yes	3	Master’s	Yes	single apartment	single
P14	26	Female	No	3	Master’s	Yes	shared apartment	single
P15	23	Female	Yes	4	Master’s	Yes	single apartment	single
P16	22	Female	No	5	Bachelor’s	Yes	shared apartment	in a relationship
P17	27	Female	Yes	4	Master’s	No	living with partner	in a relationship
P18	23	Male	No	5	Master’s	No	shared apartment	single
P19	24	Female	Yes	3	Master’s	Yes	shared apartment	in a relationship
P20	23	Male	Yes	3	Bachelor’s	No	dormitory	in a relationship

## Data Availability

The data are available from the corresponding authors on request.
